# Breaking the cycle of emotional flooding: the protective role of women’s emotional intelligence in couple’s conflict

**DOI:** 10.3389/fpsyg.2023.1217513

**Published:** 2023-08-01

**Authors:** María Berenguer-Soler, Álvaro García del Castillo-López, David Pineda

**Affiliations:** ^1^Department of Health Psychology, Miguel Hernández University of Elche, Elche, Spain; ^2^Forensic Psychology Unit, Health Psychology Department of the Center of Applied Psychology of the Miguel Hernández University of Elche, Elche, Spain

**Keywords:** perceived emotional intelligence, conflict, women, positive conflict resolution styles, emotional flooding

## Abstract

**Introduction:**

One of the most damaging aspects, both for people’s well-being and for close relationships, is conflict. Beyond different stressors, the emotions evoked, their regulation and an appropriate conflict resolution strategy will reduce negative consequences. Emotional Intelligence facilitates social relationships, but little applied research has been done on the relationship with couple conflict and emotional flooding, particularly from the perspective of women. Therefore, the present study analyzes the role of Perceived Emotional Intelligence (PEI) and the mediating effect of Positive Conflict Resolution strategies (PCR) in couples’ conflicts from women’s perspective, examining its effect on Emotional Flooding (EF) and Satisfaction.

**Methods:**

Through a cross-sectional design, the relationships between variables were analyzed using group comparisons and means of a structural equation model (SEM) in a sample of 692 women.

**Results:**

Significant differences were found between the groups by age, length of relationship, and motherhood. The SEM revealed a good fit. PEI predicted 71.8% of the variance in EF and 35% in Satisfaction through PCR and Conflict.

## 1. Introduction

Humans are social creatures who need to interact with others to form networks that act as emotional and social support systems (Rodríguez and Díaz, 2013). As a result, there is a potential for conflict in all human relationships, which increases when there is greater proximity, shared time and intimacy with the other person, as is the case in romantic relationships (Isaza, 2011; Kieslich and Steins, 2022; Shrout et al., 2023). Couple relationships have evolved considerably over recent decades. Since the late twentieth century, relationships have become increasingly egalitarian, but less stable and long-lasting, resulting in more breakups or patterns of breakup and renewal (García and de Oliveira, 2007; Monk et al., 2018). Gender stereotypes traditionally ascribed to men and women, at least in Western cultures, suggest that women are more emotional than men (Fischer, 2000; Chentsova-Dutton and Tsai, 2007), in terms of awareness, expression, sensitivity, or lability (Diener et al., 1985; Grossman and Wood, 1993; Kring and Gordon, 1998; Else-Quest et al., 2012; Fernández-Aguilar et al., 2018). Although emotions have been the subject of research for decades, evidence on how Conflict in couples affects Emotional Flooding and Satisfaction is scarce, especially from the unique female perspective and the use of Positive Conflict Resolution strategies. In order to fill this gap, the present study was developed, aimed to analyze the relationships between all these variables and the proposed role of the Perceived Emotional Intelligence as a protective construct.

### 1.1. Emotion manners, emotion matters

Emotions play a crucial role in relationship dynamics, with their elicitation and management being essential factors that can significantly influence conflict outcomes (Kopystynska et al., 2017; Kieslich and Steins, 2022). Emotions are complex human states that involve cognitive, physiological, and behavioral components (Kolb et al., 2019). These components encompass subjective states and physiological responses that prepare the organism to respond to stimuli. Evidence in the literature suggests that people maintain a certain degree of control over their emotions, using various strategies to regulate how and when these emotions are experienced. In doing so, individuals can monitor, evaluate, and modify the processes involved in the genesis of their emotions, allowing them to modulate the intensity and expression of these emotions (Salovey and Mayer, 1990; Mayer et al., 1999; Pineda et al., 2018). This ability to control and use emotions to guide thought and behavior is what Salovey and Mayer (1990) defined as Emotional Intelligence (EI), distinguishing between the processing of affective information at intrapersonal and interpersonal levels. EI has been shown to enhance people’s ability to cope with stressors and conflict situations of high emotional intensity (Ruiz-Mamani et al., 2022) and is related to important social functioning variables such as attachment style and family cohesion (Cócola, 2022). Moreover, recent reviews have highlighted the role of EI in several health and social scenarios; improving the quality of life of breast cancer survivors (Durosini et al., 2022), enhancing EI in patients with panic disorder to improve their flexibility in emotion regulation (Oussi et al., 2023), or in individuals with social anxiety disorder (Rozen and Aderka, 2023). In the specific context of couple relationships, EI has been shown to be related to several variables. In a recent systematic review and meta-analysis, Jardine et al. (2022) found a positive and significant correlation between EI and romantic relationship satisfaction. In addition, and focusing on women, interventions aimed at working on and increasing EI in married couples are associated with improved marital satisfaction, sexual quality of life, and psychological well-being. (Milani et al., 2020). In a multiple study, Alonso-Ferres et al. (2019) found gender differences in coping with couple conflict and a mediating role of EI in the adoption of adaptive responses, leading to greater psychological well-being and higher relationship satisfaction.

As shown, EI has a significant impact on individuals’ lives and reactions (Zeidner and Kaluda, 2008), and an intelligent use of emotions leads to an appropriate handling of both emotions and relationships (Goleman et al., 2002). Beyond individual differences, traditional socialization of boys and girls according to gender produces different emotional experiences in terms of emotional intensity, strength, clarity, and instability (Bailen et al., 2019). The need for researchers to consider the gender perspective to break down some of the traditional stereotypes and broaden the field of study regarding women and emotions has been widely accepted by the scientific community, especially in social and couple situations where emotional identification, expression, and regulation are essential, such as in the psychological context of domestic violence (Ferrer and Bosch, 2005; Pineda et al., 2023). Indeed, research has shown that in the context of couples, women’s emotional intelligence is negatively related to the propensity to initiate and maintain a relationship with an abusive partner (Ambler et al., 2023). Therefore, in close relationships, the deepest emotions and feelings are shared; therefore, couples must be able to understand, talk about and manage them to ensure a healthy relationship (Batool and Khalid, 2012). Perceived Emotional Intelligence (PEI) is the perception of one’s emotional abilities as measured by self-report (Salovey et al., 2002). Following the model developed by Mayer and Salovey (1997), (for a more in-depth theoretical review see Fernández-Berrocal and Extremera, 2006) and the ensuing debate on how to measure EI, the term PEI has been proposed to refer to individual meta-knowledge about one’s emotional abilities rather than actual capacity, measuring fundamental aspects of emotional self-awareness such as perceived attention to one’s own emotional states (Emotional Attention), perceived understanding of these states (Emotional Clarity), and perceived ability to regulate these states (Emotional Repair; Extremera and Fernández-Berrocal, 2005; Salguero et al., 2012). In romantic relationships, emotional awareness and the ability to identify and regulate emotions have a significant impact on the well-being of couples (Fishbane, 2011). PEI has been positively related to adaptive coping and problem solving, especially when considering the Emotional Clarity factor (Augusto et al., 2008). Furthermore, those capable of perceiving, assimilating, and regulating their emotions, as well as appropriately interpreting those of others, are more resilient and more satisfied with life (Veloso-Besio et al., 2013). Therefore, PEI can be crucial in preventing conflict situations in couples by effectively identifying and regulating emotions that can cause problems in the relationship. Positive conflict resolution styles have been associated with effective anger regulation, which helps avoid negative unregulated emotions that drive conflict (Rivers et al., 2007).

### 1.2. The straw that broke the camel’s back

High emotional arousal in interpersonal conflict situations can lead to “flooding,” a state of overstimulation, overwhelm and cognitive disorganization (Malik et al., 2020). This term was first introduced by behavior psychologists analyzing the treatment of anxiety due to immediate and prolonged exposure to feared stimuli (Rachman, 1966). However, it was (Gottman, 1993a,b) who first used the term Emotional Flooding (EF) to refer to couple relationships in which hard-to-control feelings of emotional unrest and overload affect sensory perception and proper cognitive functioning. Emotions guide our decisions along with the rational mind, and the thinking brain moderates their expression, except in situations of EF, when emotions overwhelm reason and the emotional brain takes complete control of the situation (Goleman, 1996). EF focusses on how an individual experiences a situation of anger and negative emotions, where their partner loses control, and how this affects the outcome. In most cases, it leads to aggressive behavioral responses, as described by Braiker and Kelley (1979) in their model described below. When on a regular basis either member of the couple feels emotionally flooded due to the attitude of his/her partner, any action carried out by said partner may be negatively interpreted, leading to intensely inappropriate reactions and negative feelings that will inhibit problem-solving (Malik et al., 2020). As time passes, the individual may feel that talking and trying to solve the problems are useless, resulting in the potential search for relief from these negative feelings in a parallel or isolated manner. This may result in an increased distancing between partners, who may ultimately decide to separate (Gottman, 1993b).

As Sotskova et al. (2015) point out, flooding is a relatively new concept in the emotion literature, but we can find theoretical similarities with aspects of emotional sensitivity. Furthermore, EF understood as the loss of rational control following a flood of intense negative emotions that leads the person to engage in irrational behaviors (Penberthy et al., 2018) can trigger unexpected and emotionally charged responses such as aggressive behaviors (Braiker and Kelley, 1979; Gómez-Leal et al., 2022) escape or withdrawal behaviors (Gottman, 1993b; Malik et al., 2020), which would ultimately have a negative impact on couple satisfaction. In this sense, aggression and impulsivity have shown indirect and significant effects with EI (Coccaro et al., 2016). Consequently, people with higher EI would be able to adopt more positive behavioral styles in stressful situations like PCR, avoiding both aggressive and dysregulated negative emotional behaviors and conflict. Therefore, this work aims to deepen the analysis between these variables, assuming that people with high emotional self-awareness, who are better able to regulate their negative emotions, will end up using more PCR when arguing with their couples and, as a result, will be less involved in conflict situations.

### 1.3. Not today: positive conflict resolution strategies to avoid conflict

Couple relationships differ from other relationships given the presence of certain unique processes and expectations such as fidelity and emotional and romantic exclusivity (Vidal et al., 2012). One of the most damaging aspects, both for people’s well-being and for the relationship, is conflict (García and Umberson, 2019). Conflicts are disagreements that can be constructive for the relationship, depending on the strategies employed to resolve them (Cascón, 2006). In the context of Social Exchange Theory (Thibaut and Kelley, 1959; Blau, 1964; Burns, 1973; Homans, 1974), Braiker and Kelley (1979) develop their model of dyadic close relationship in which they underline the emotion-conflict relation according to the intensity. Thus, with a high intensity of interpersonal conflict, we will find a high level of emotional activation, leading the subject to resolve the conflict in the short term through the use of two strategies; escape or exaggerated behavioral response (e.g., aggressive behavior against the partner). Researchers have found significant differences when examining conflict resolution strategies used by men and women in their relationships (Black, 2000; Alexander, 2001; Laca et al., 2006) as well as in other areas such as education (Skordoulis et al., 2020; Galindo-Domínguez et al., 2022). In general, women tend to use more positive strategies, such as cooperation-based conflict resolution styles, compared to men who typically rely on more aggressive styles (Ma, 2005; Garaigordobil and Maganto, 2011; Garaigordobil et al., 2016). The ability to resolve conflicts is therefore a key element in the development of social relationships, and is particularly important in sentimental relationships, where disagreements and arguments often arise which, if not properly resolved, can lead to insecurity, deception or dissatisfaction, both sexual and with the partner (de Wied et al., 2007; Quesada, 2020). In addition, certain couple characteristics such as age or length of relationship, which may influence both the frequency and intensity of conflict, have been shown to be associated with increased levels of emotional partner violence (Juarros-Basterretxea et al., 2022; Rojas-Solís and Romero-Méndez, 2022). Throughout his research, Gottman has also been interested in analyzing the behavior that occurs in conflict situations between couples (Gottman, 1993b, 1994), conflicts identified as the “four horsemen of the apocalypse” (criticism, defensiveness, contempt, and stonewalling), which are essentially negative conflict resolution styles given their impact on personal well-being and relationship satisfaction. Therefore, in line with this approach, in the present research, we expect to find that the opposite strategies, i.e., Positive Conflict Resolution strategies (PCR), lead to fewer and less intense conflicts with the partner.

On the basis of the evidence described above, we conducted the present research in an all-female sample with a 2-fold aim. On the one hand, we sought to extend the literature on women’s relationships by exploring group differences by age and relationship length on the endogenous variables of PCR, Conflict, EF, and Satisfaction. We also wanted to see if having children had any impact on Conflict and Satisfaction. On the other hand, we want to analyze the relationship between PEI, PCR, Conflict, EF, and Satisfaction by building an explanatory model using structural equations in this sample of women. To this end, the following hypotheses were formulated:

*H1*: PEI is direct and positively related to PCR.

*H2*: PEI is direct and negatively related to Conflict.

*H3*: PCR is direct and negatively related to Conflict.

*H4*: Conflict is direct and positively related to EF.

*H5*: Conflict is direct and negatively related to Satisfaction.

*H6*: EF is direct and negatively related to Satisfaction.

## 2. Materials and methods

### 2.1. Design

**Figure 1:**
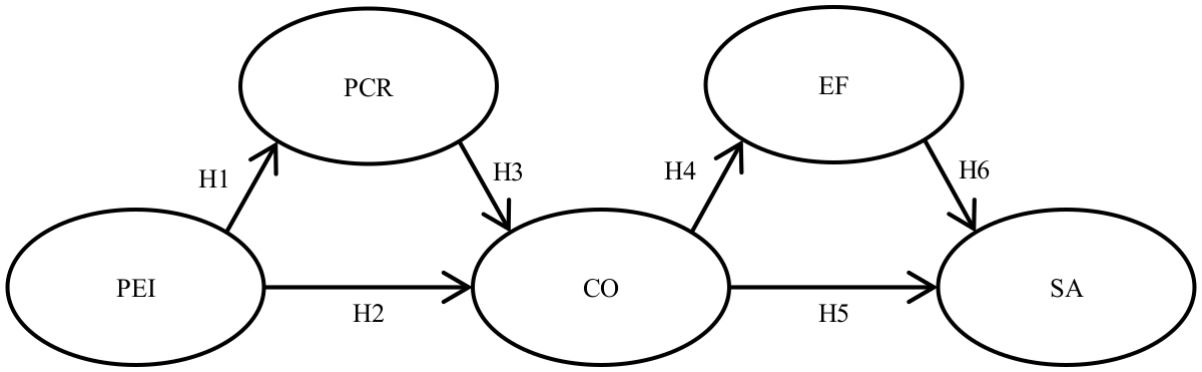
Hypothetical model of causal relationships and hypotheses. PEI, perceived emotional intelligence; PCR, positive conflict resolution strategies; CO, conflict; EF, emotional Flooding; and SA, satisfaction.

This study followed a cross-sectional design, with participants being recruited through an online questionnaire and relying on non-probabilistic, snowball sampling that was distributed across distinct social media directed at Spanish residents. The hypothetical model based on the theory, together with the proposed hypotheses, is shown in Figure 1.

### 2.2. Participants and procedure

Participation in this study was voluntary and no compensation or payment was given to the participants. After an initial literature review and selection of instruments, an online questionnaire was created in Google Docs and the link was distributed through several social networks such a Twitter, Facebook. and Instagram. Participants were asked to share the link with friends and acquaintances. The study objectives were explained along with the potential benefits of participation. Informed consent was requested from the participants to process their data. A total of 825 responses were received. As the purpose of the study was to analyze the functioning of the variables from a gender perspective, only responses from female participants who were currently or previously in a relationship with a heterosexual partner were selected. The sample size exceeded the minimum required considering the number of latent constructs and items (Hair et al., 2010) This study is part of a larger research project and was conducted following the Declaration of Helsinki guidelines. Research ethical approval for the study was granted by the Research Ethics and Integrity Committee of Miguel Hernández University, Spain (DPS.AGL.01.21).

The final sample consisted of 692 women with a mean age of 31.76 years (*SD* = 7.99). Of these, 64.6% were involved in a couple relationship, 21.7% were married, 11.0% single, 2.3% divorced, and 0.4% widowed. Of the selected sample, 28.0% had children. To make comparisons between groups, the sample was divided into three age groups and four groups according to the length of the relationship. Age groups were created following the recommendations of Martín (2005): young adults (up to 39 years), middle-aged (40–49 years), and older adults (>50 years). The groups by relationship length were divided into four categories according to a statistical distribution criterion of equal percentiles based on scanned cases with three cut-off points: less than 3 years, between 3 and 7 years, between 7 and 11 years, and more than 11 years. A total of 32.1% of the sample had been in a relationship for 3 years or less, 29.9% between 3.1 and 7 years, 18.4% between 7.1 and 11 years, and 19.7%, for over 11.1 years. Most of the participants had studies at the university or master’s level studies (83.8%).

### 2.3. Measures

A sociodemographic data questionnaire was used in this study. It was an *ad hoc* questionnaire that recorded age, civil status, sexual orientation, number of children, and level of education. In addition, a Spanish version of the Trait Meta-Mood Scale (TMMS-24) by Salovey et al. (1995), the Scale of Emotional Flooding in Couple Relationships (Berenguer, 2019), the Spanish version of the Conflict Resolution Style Inventory (CRSI; Kurdek, 1994), the Spanish version of the Kansas Marital Satisfaction Scale (KMS; Schumm et al., 1983), and an *ad hoc* questionnaire measuring conflict in couples were used.

#### 2.3.1. Trait meta-mood scale

Perceived emotional intelligence was measured using the validated Spanish version by Fernández-Berrocal et al. (2004) of the TMMS-24 (Salovey et al., 1995). This instrument collects meta-knowledge from individuals on how they identify and handle their moods, emotions, and feelings, using a five-point Likert scale. The responses ranged from 1 (*strongly disagree*) to 5 (*strongly agree*). Studies that analyze this factorial structure have revealed the existence of three dimensions or factors: (1) Emotional Attention: Consisting of items that comprise the capacity to pay attention to feelings and emotions (e.g., “I pay a lot of attention to my feelings”); (2) Emotional Clarity: Consisting of items indicating the ability to understand emotional states and to discriminate between different emotions and feelings (e.g., “I am clear about my feelings”); and (3) Emotional Repair: Consists of items determining the ability to appropriately regulate one’s emotional states (e.g., “although I sometimes feel sad, I usually have an optimistic outlook”). The results are classified into three groups, according to the authors’ recommendations: low, good, and excellent skills in factors 2 and 3, and low, good, and excessive skills in factor 1. Prior studies have analyzed the scale’s internal consistency and validity (Górriz et al., 2021; Ruiz-Mamani et al., 2022). Its reliability for the sample was high (α = 0.94) as well as that of the factors (α_1_ = 0.90, α_2_ = 0.92, α_3_ = 0.90).

#### 2.3.2. Conflict resolution style inventory

The PCR used by the couple was measured using the positive problem-solving dimension of Conflict Resolution Style Inventory (CRSI) according to the participants’ perspective in an adapted version that was validated in Spanish (Arenas, 2014). It consisted of four items with a five-point Likert-like response ranging from 1 (*almost never*) to 5 (*almost always*) measuring the degree to which the individual used positive problem-solving strategies (i.e., negotiation strategies) in response to conflicts, with questions about the frequency of situations such as “sitting *down and discussing our differences constructively*.” Prior studies have analyzed the internal consistency and validity of the inventory (Arenas, 2014; Arenas and Hidalgo, 2016). Its reliability for the sample was acceptable, as was the case in previous studies (α = 0.72).

#### 2.3.3. Scale of emotional flooding in couple relationships

Emotional Flooding was measured using the Scale of Emotional Flooding in Couple Relationships (EDEP) by Berenguer (2019). EF regarding couple relationships refers to the state in which the individual feels overwhelmed by the circumstances surrounding said relationship, having a negative perception of the emotions of the other member of the couple. The instrument consists of 18 items with a five-point Likert-type response scale ranging from 1 (*completely disagree*) to 5 (*completely agree*). It is based on four factors, according to the original proposal made by Gottman (1993a): Susceptibility or hypersensitivity to negative attitudes, Unjustified or disproportionate Anger, Motivation to Run Away, and Emotional Self-Regulation. For this study, all factors were used. The Susceptibility factor is measured using items such as “*my partner’s negative attitudes overwhelm me*”; the Unjustified Anger factor was measured using items such as “*I get overly upset when I argue with my partner*,” the Motivation to Run Away factor was measured using items such as “*when we argue I want to escape and have nothing more to do with the relationship*” and the Emotional Self-Regulation factor was measured using items such as *“I find it difficult to control my anger when arguing with my partner.”* The reliability of the scale in the sample was high (α = 0.92) as was that of the factors (α_1_ = 0.83, α_2_ = 0.90, α_3_ = 0.91, α_4_ = 0.87).

#### 2.3.4. Kansas marital satisfaction scale

Satisfaction was measured using the validated Spanish version by Montes-Berges (2009) of the Kansas Marital Satisfaction Scale (Schumm et al., 1983). This is a 4 item-scale evaluating satisfaction with the partner using a seven-point Likert-type response scale ranging from 1 (*completely dissatisfied*) to 7 (*completely satisfied*). Satisfaction is measured using items such as “*How satisfied are you with your current relationship or marriage?.”* The reliability of the scale in the sample was high (α = 0.89).

#### 2.3.5. Conflict

An *ad hoc* questionnaire based on the multidimensional model of emotion and Braiker and Kelley (1979) was used to measure conflict with the partner, measured by means of 3 items assessing frequency, valence, and arousal: (a) *How often do you and your partner argue?* (b) *When my partner and I disagree, we discuss it calmly,* and (c) *Arguing with my partner is very intense*. Responses were measured on a five-point Likert-type scale ranging from 1 (*completely disagree*) to 5 (*completely agree*). The reliability of the scale in the sample was acceptable (α = 0.70).

### 2.4. Data analysis

The SPSS 18.0, AMOS 23.0, and JASP 0.17.1 statistical packages were used for descriptive analysis, correlations, factorial analyses, and between-groups comparisons. Data were preliminarily screened for outliers and errors (not detected). For correlations, Spearman’s rho coefficient was used. The normality of the variables was verified with the Kolmogorov–Smirnov test and homoscedasticity was examined using Levene’s test for equality of variance. Due to the lack of assumptions required for parametric tests such as normality and symmetry, Kruskal-Wallis H and Mann–Whitney U tests were used to compare groups. For all of these tests, an alpha level of 0.05 was used. To correct the possible error resulting from the 5% threshold, the Bonferroni correction was used. The *post hoc* Dunn’s adjustment test was performed to examine significant differences between groups. Effect sizes were calculated using r and Cohen’s *d*.

Confirmatory factorial analyses (CFA) were used to assess the psychometric properties of the instruments, as well as their factorial structure. Varimax rotation was used for the extraction of the main components. Estimation, identification, assessment, and re-specification of the structural equation model (SEM) were performed in AMOS. A variance–covariance matrix was used as well as maximum likelihood estimation. When estimating the parameters that define the factors, as well as the relations between variables, recommendations of Bentler and Chou (1987) were considered with respect to sample size.

For path analysis, SEM was used. Direct and indirect effects were calculated using a bootstrap technique to determine significance. A bootstrap sample of 5,000 was used. Significance was assessed using the bias-corrected percentile method with a 95% confidence interval. A multicollinearity test was performed with negative results. The goodness of fit parameters were estimated using a maximum likelihood estimation model. Given the lack of consensus regarding the concept of fit, various indices were considered simultaneously (Tanaka, 1993) to analyze the model’s fit: the discrepancy between the Chi-squared distribution and the degrees of freedom (χ2/df, values lower than 5 are considered correct), the root mean square error of approximation (RMSEA, a value equal to or less than 0.05 indicates a good fit). Values equal to or less than 0.08 can also be considered to correspond to reasonable errors of approximation (Jöreskog, 1993), the normed fit index (NFI, values above 0.90 are interpreted as an acceptable fit; Byrne, 1994, 2010; Marsh and Grayson, 1995), the Tucker-Lewis index (TLI, values above 0.90 indicate an acceptable fit (Bentler and Bonett, 1980), the comparative fit index (CFI, values equal to or greater than 0.90 indicate a good fit; Byrne, 1994, 2010; Bentler, 2005), the goodness-of-fit index (GFI, values above 0.90 are considered an acceptable fit; Marsh and Grayson, 1995), and the adjusted goodness-of-fit index (AGFI, values above 0.90 are considered an acceptable fit; Jöreskog and Sörbom, 1981, 1984, 1986; Hoyle, 1995).

## 3. Results

### 3.1. Descriptive data and correlations

Table 1 shows the descriptive statistics of the scales completed by participants according to relationship length and age.

**Table 1:** Descriptive statistics by age group and relationship length.

	Age			Relationship length			
	YA	MA	OA	<3	3–7	7–11	+11
	*n* = 580*M*(*SD*)	*n* = 85*M*(*SD*)	*n* = 27*M*(*SD*)	*n* = 222*M*(*SD*)	*n* = 207*M*(*SD*)	*n* = 127*M*(*SD*)	*n* = 136*M*(*SD*)
EA	27.17 (6.22)	26.26 (7.04)	24.70 (7.39)	–	–	–	–
EC	27.28 (6.57)	27.25 (6.93)	26.89 (5.98)	–	–	–	–
ER	25.32 (6.43)	25.27 (6.78)	27.89 (6.87)	–	–	–	–
PCR	15.74 (2.66)	15.33 (2.83)	14.56 (2.50)	15.87 (2.76)	16.04 (2.67)	15.55 (2.61)	14.75 (2.45)
EF	42.12 (12.76)	45.86 (13.24)	48.79 (10.87)	40.96 (13.75)	43.01 (12.79)	42.65 (12.55)	45.84 (11.16)
SA	12.97 (1.90)	12.28 (1.94)	11.63 (2.24)	12.95 (2.09)	12.98 (1.71)	13.08 (1.86)	12.19 (1.99)
CO	6.74 (1.82)	7.38 (1.94)	7.26 (1.32)	6.59 (1.76)	6.70 (1.84)	6.94 (1.86)	7.35 (1.83)

YA, young adults; MA, middle-aged adults; OA, older adults; EA, emotional attention; EC, emotional clarity; ER, emotional repair; PCR, positive conflict resolution strategies; EF, emotional flooding; SA, satisfaction; CO, conflict. Relationship Length expressed in years.

Table 2 shows the correlations between the variables.

**Table 2:** Spearman’s correlations.

	1	2	3	4	5	6
1. EA	–					
2. EC	**0.542**	–				
3. ER	**0.445**	**0.630**	–			
4. PCR	**0.239**	**0.385**	**0.271**	–		
5. EF	0.051	**−0.268**	**−0.232**	**−0.388**	–	
6. SA	0.074	**0.216**	**0.128**	**0.346**	**−0.355**	–
7. CO	−0.010	**−0.141**	**−0.160**	**−0.364**	**0.560**	**−0.385**

EA, emotional attention; EC, emotional clarity; ER, emotional repair; PCR, positive conflict resolution strategies; EF, emotional flooding; SA, satisfaction; CO, conflict. In bold significant coefficients. *p* < 0.01.

### 3.2. Comparison of groups

**Figure 2:**
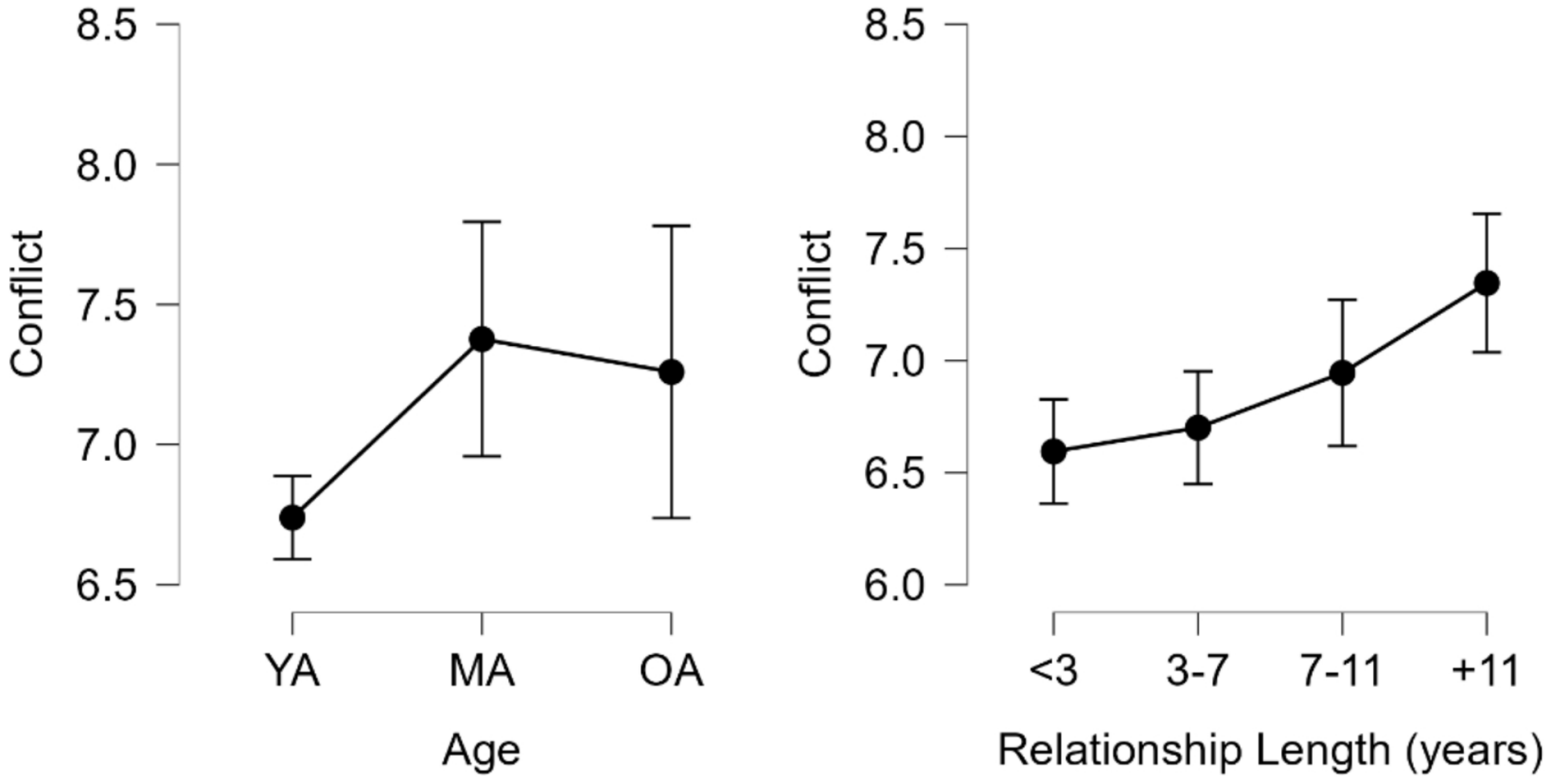
Conflict scores by age and relationship length. YA, young adults; MA, middle-aged adults; and OA, older adults.

Significant differences in Conflict by age were found (Figure 2) between the Young Adults (YA, *M* = 6.740, *SD* = 1.819) and Middle-Aged adults (MA, *M* = 7.376, *SD* = 1.939) groups H (2) = 11.914, *p* < 0.05, with higher conflict scores in the MA group, with a moderate effect size *d* = 0.350, 95% CI = 0.071–0.630. By relationship length, significant differences were found between <3 (*M* = 6.595, *SD* = 1.756) and + 11 (*M* = 7.346, *SD* = 1.823) groups as well as between 3–7 (*M* = 6.700, *SD* = 1.835) and + 11 groups H = 15.32 (3), *p* < 0.001, with moderate effects sizes *d* = 0.415, 95% CI = 0.125–0.704 and *d* = 0.356, 95% CI = 0.063–0.649, respectively.

**Figure 3:**
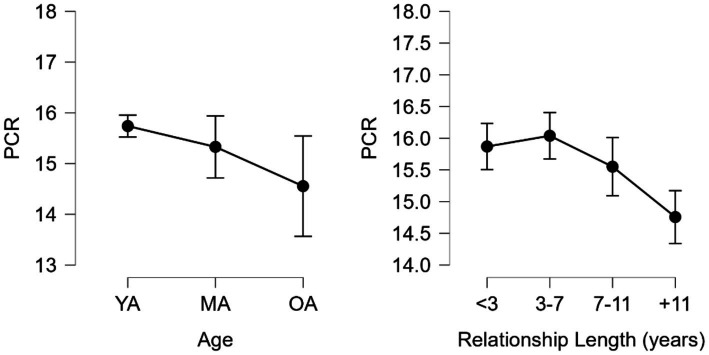
Positive conflict resolution strategies (PCR) by age and relationship length. YA, young adults; MA, middle-aged adults; and OA, older adults.

In terms of PCR, significant age differences were found (Figure 3) between the YA (*M* = 15.740, *SD* = 2.656) and Older Adults (OA, *M* = 14.556, SD = 2.501) groups H (2) = 7.264, *p* < 0.05, with moderate effect size *d* = 0.443, 95% CI = 0.030–0.916. By relationship length, significant differences were found H (3) = 20.080, *p* < 0.001 between <3 (*M* = 15.869, *SD* = 2.758) and + 11 (*M* = 14.757, *SD* = 2.454) groups, 3–7 (*M* = 16.039, *SD* = 2.666) and + 11 groups, as well as 7–11 (*M* = 15.551, *SD* = 2.609) and + 11 groups, with moderate effects sizes *d* = 0.420, 95% CI = 0.131–0.710, *d* = 0.484, 95% CI = 0.190–0.778 and *d* = 0.300, 95% CI = 0.027–0.627, respectively.

**Figure 4:**
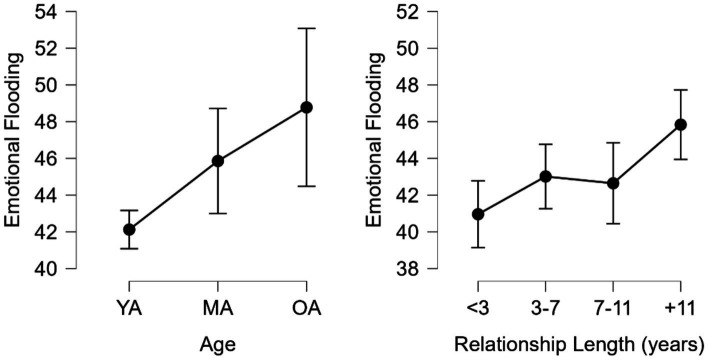
Emotional flooding by age and relationship length. YA, young adults; MA, middle-aged adults; and OA, older adults.

Regarding EF (Figure 4) significant differences were found H (2) = 13.042, *p* < 0.001 between YA (*M* = 42.124, *SD* = 12.762) and MA (*M* = 45.859, *SD* = 13.238) groups as well as YA and OA (*M* = 48.778, *SD* = 10.868) groups, with a moderate effect size *d* = 0.293, 95% CI = 0.013–0.572 and medium effect size *d* = 0.522, 95% CI = 0.048–0.995, respectively. By relationship length, significant differences were found H (3) = 16.407, *p* < 0.001 between <3 (*M* = 40.959, *SD* = 13.748) and + 11 (*M* = 45.838, *SD* = 11.157) groups, 3–7 (*M* = 43.014, *SD* = 12.785) and + 11 groups as well as 7–11 (*M* = 42.646, *SD* = 12.554) and + 11 groups, with moderate effect sizes *d* = 0.382, 95% CI = 0.093–0.672, *d* = 0.221, 95% CI = 0.071–0.514 and *d* = 0.250, 95% CI = 0.077–0.577, respectively.

**Figure 5:**
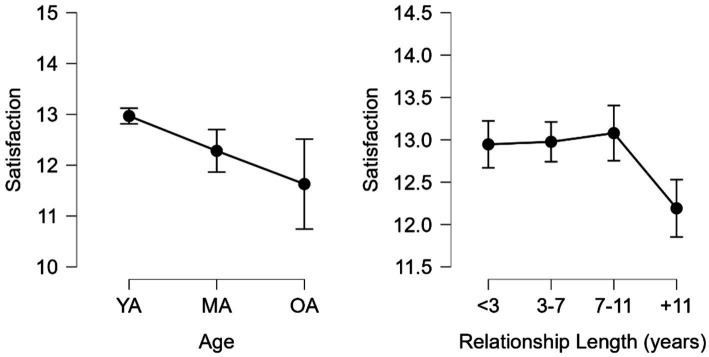
Satisfaction by age and relationship length. YA, young adults; MA, middle-aged adults; and OA, older adults.

Satisfaction also was significant when comparing groups (Figure 5). By age, significant differences were found between YA (*M* = 12.967, *SD* = 1.902) and MA (*M* = 12.282, *SD* = 1.937) groups H (2) = 18.163, *p* < 0.001 with moderate effect size *d* = 0.357, 95% CI = 0.077–0.636 as well as between YA and OA (*M* = 11.630, *SD* = 2.239) with a medium effect size *d* = 0.697, 95% CI = 0.222–1.171. By relationship length, significant differences were found H (3) = 20.230, *p* < 0.001 between <3 (M = 12.946, *SD* = 2.094) and + 11 (*M* = 12.191, *SD* = 1.994) groups with moderate effect size *d* = 0.392, 95% CI = 0.103–0.682, 3–7 (*M* = 12.976, *S*D = 1.711) and + 11 groups with moderate effect size *d* = 0.408, 95% CI = 0.115–0.702, as well as 7–11 (*M* = 13.079, *SD* = 1.859) and + 11 groups, with moderate effect size *d* = 0.462, 95% CI = 0.133–0.790.

Finally, a significant difference was found when comparing the groups according to whether they had children or not, with higher levels of conflict found in the group of couples with children (*Mdn* = 7, *n* = 194) *U* = 37137.000, *z* = 4.795, *p* < 0.001, *r* = 0.18 and higher scores on Satisfaction in the group without children (*Mdn* = 13, *n* = 498) *U* = 38926.500, *z* = 4.134, *p* < 0.001, *r* = 0.16.

### 3.3. Building an explanatory model with structural equations

**Figure 6:**
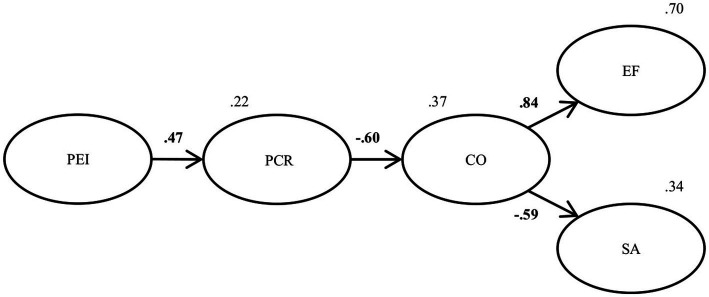
Simplified final model with standardized coefficients. PEI, perceived emotional intelligence; PCR, positive conflict resolution strategies; CO, conflict; EF, emotional flooding; and SA, satisfaction. In bold significant coefficients. *p* < 0.001.

An exploratory factor analysis was conducted to determine the behavior of the conflict questionnaire items and their construct validity. A confirmatory factor analysis was conducted with all other variables included in the model and all the scales retained the original item and factor structure. After the first analysis, the relationship between PEI and Conflict (H2) *b* = 0.05, *p* = 0.240 and EF and Satisfaction (H6) *b* = 0.000, *p* = 0.992 were found to be insignificant and therefore dropped from the model, so H2 and H6 were not supported. The definitive, simplified SEM along with the standardized structural coefficients is shown in Figure 6. The SEM revealed a good fit with the data χ^2^(2, *N* = 692) = 3.373, *p* < 0.001; RMSEA = 0.059, 95% CI: 0.052, 0.065; NFI = 0.923; TLI = 0.931; CFI = 0.944; GFI = 0.941 and AGFI = 0.918. The direct effect of PEI on PCR was found to be positive and significant *b* = 0.032, *p* < 0.001, supporting H1. The direct effect of PCR on Conflict was found to be negative and significant *b* = −0.711, *p* < 0.001, supporting H3. The direct effect of Conflict on EF was found to be positive and significant *b* = 4.465, *p* < 0.001, supporting H4. Finally, the direct effect of Conflict on Satisfaction was found to be negative and significant *b* = −0.791, *p* < 0.001, supporting H5.

In summary, all hypotheses except H2 and H6 were supported and a significant indirect effect was found between PEI and Conflict, EF and Satisfaction through the mediators (Table 3), explaining 70% of the variance in EF and 34% of the variance in Satisfaction.

**Table 3:** Mediation analysis summary.

	IE	C.I.		*p*
		LB	UB	
PEI⤏PCR⤏CO	−0.023	−0.029	−0.018	0.000
PEI⤏PCR⤏CO⤏EF	−0.102	−0.133	−0.075	0.000
PEI⤏PCR⤏CO⤏SA	0.081	0.054	0.117	0.000

IE, indirect effect; PEI, perceived emotional intelligence; PCR, positive conflict resolution strategies; CO, conflict; EF, emotional flooding; SA, satisfaction. *p* < 0.001.

## 4. Discussion

The aim of the present study was to better understand the relationship between PEI and Conflict in a sample of women, analyzing the role of PCR, EF and Satisfaction. To this end, the relationships between these domains were examined using a full structural equation model. Differences between groups by having children, age, and relationship length were also examined, with the aim of extending the evidence in the women’s literature on couples, and significant results were found. Our results showed an escalation in levels of conflict as a function of age and relationship length, with women aged 40–49 (middle-aged) having higher levels of conflict than younger women. Similarly, women who had been in a relationship for more than 11 years had higher levels of conflict with their partner than women who had been in a relationship for less than 3 years or between 3 and 7 years. This finding is consistent with previous research showing that older married women tend to report more anger than middle-aged adults in an experiment involving disagreements with their husbands (Smith et al., 2009). Although the present study did not find significant differences in the over 50s group, as found in the study by Smith et al., the variation between groups is equivalent and a significant effect of age in couple conflict was found. This idea is supported by the reality of life course transitions in relationships, where significant changes occur when people decide to share or buy a house, get married or have children (Vanderbilt and Solomon, 2022). These decisions, influenced by age and relationship length, could lead to an increase in both the frequency and intensity of arguments within the couple (Booth et al., 2016). Our results also showed a significant negative relation between age and use of PCR when comparing groups, so that the higher the age, the lower the number of PCRs used in conflicts with the partner. This trend of decreasing PCR use among women as a function of age is reflected in previous studies that have analyzed the impact of external stressors on relationship quality and satisfaction, such as the case of COVID-19 quarantine and its impact on couple relationships (Işık and Kaya, 2022). Characteristics of emotional aging have been extensively described (Carstensen et al., 2003; Kunzmann et al., 2014; Fairfield et al., 2015; Kunzmann and Wrosch, 2018), along with EI’s relationship with important psychological and behavioral domains in older adults, including well-being (Chen et al., 2016; Delhom et al., 2017; Buedo-Guirado et al., 2021), addictions (García del Castillo et al., 2013), resilience (Meléndez et al., 2019), and quality of life (Luque-Reca et al., 2018). However, the effect of EF as a function of age has received less attention in the literature. Previous research has shown a significant difference in affective intensity by gender, with women scoring higher, but only in younger groups (Diener et al., 1985). When analyzing valence and arousal of both positive and negative emotions in a mood induction scenario, significant age differences were found in older adults exposed to stimuli eliciting disgust, fear, anger, and sadness (Fernández-Aguilar et al., 2018). Given that conflict situations with the partner are generated in an environment of negative emotions and that EF is an emotional state characterized by the presence of these emotions, the results of our study are consistent in finding an increase in EF as a function of age. We also found significant differences in Conflict, PCR, and EF when comparing groups by relationship length. Shorter relationships in our sample had lower conflict scores than longer ones, used more PCR and had lower scores on EF, a tendency consistent with the literature analyzing the effects of relationship length on several key areas of close and intimate interpersonal functioning related to conflict (Totenhagen et al., 2016; Kuncewicz et al., 2021; Vanderbilt and Solomon, 2022). The results regarding satisfaction with the partner as a function of age, relationship length, and having or not having children are consistent with the literature. Parenting is related to lower marital satisfaction, even more if we speak of mothers (Twenge et al., 2003). This may be explained by the strains of parenthood (Hansson and Ahlborg, 2016), parenting stress and marital intimacy (Chester and Blandon, 2016), or even a decrease in sexual satisfaction (Vasconcelos et al., 2021).

The results of the SEM confirmed the role of PCR and Conflict as mediators explaining the relationship between PEI, EF, and Satisfaction in our sample. The need to apply appropriate conflict resolution strategies to positively resolve couple relationship problems has been clearly demonstrated in numerous studies (de Wied et al., 2007; Gottman and Silver, 2012; Garaigordobil et al., 2016), which is consistent with our findings. Also, the relationship between intense emotions of anger and the EF in the context of intimate partner violence and distress (Malik et al., 2020). The way couples deal with conflict has an important impact on their satisfaction. Constructive conflict behaviors, such as PCR, are associated with higher levels of partner satisfaction, whereas conflict avoidance strategies, such as withdrawal and frequency of conflict, are associated with lower levels of satisfaction (Johnson et al., 2018). On the other hand, we did not find a direct relationship between PEI and Conflict, as suggested by previous work (Christensen and Walczynski, 1997; Jardine et al., 2022; Winardi et al., 2022), but rather that the relationship was mediated by PCR. Nevertheless, this relationship is consistent with the literature examining how positive and negative strategies in conflict are related to EI (Perles et al., 2011; Sabo, 2020).

## 5. Conclusion

Couple relationships are a complex environment in which different episodes, emotions and situations occur that affect both the couple’s well-being and several individual variables. To our knowledge, this is the first study to examine the importance of Perceived Emotional Intelligence and Positive Conflict Resolution strategies in situations of partner conflict, explaining Emotional Flooding and satisfaction as outcomes from a female perspective. The findings highlight the need for adequate Perceived Emotional Intelligence and Positive Conflict Resolution strategies to prevent partner conflict, reduce the occurrence of Emotional Flooding, and increase relationship satisfaction. These results would justify the need to work on emotional competences together with Positive Conflict Resolution strategies in any couple context in order to improve interpersonal relationships, whether at the clinical, intervention, or prevention program level. In addition, we found relevant evidence to add to the women’s literature when analyzing groups. Conflict increased as a function of age and relationship length, as did Emotional Flooding, while Positive Conflict Resolution strategies and Satisfaction decreased.

### 5.1. Limitations and future research

The study’s primary participants are individuals who identify as heterosexual, are in monogamous, committed relationships—including marriage—and have been in relationships for a significant length of time. Although this selection criterion was motivated by the fact that most previous research on intimate relationships has focused on the experiences of heterosexual couples, caution should be exercised in generalizing the results. Additionally, the vast majority of the sample had higher education levels and the age sample was unbalanced, which may limit the generalisability of the findings to other social groups. To provide a more comprehensive understanding of the implications of the relationships found in the model, a longitudinal study could be conducted to analyze the functioning of couples over time and observe changes in the variables of interest. Future research could also explore the role as potential risk or protective factors in intimate partner violence.

## Data availability statement

The datasets presented in this study can be found in online repositories. The names of the repository/repositories and accession number(s) can be found at: https://osf.io/ng5ku/?view_only=f450eb6faad24e7ebe4db2b7a9e0ac4c.

## Ethics statement

The studies involving human participants were reviewed and approved by Research Ethics and Integrity Committee of Miguel Hernández University, Spain (DPS.AGL.01.21). The patients/participants provided their written informed consent to participate in this study.

## Author contributions

AG and MB-S designed the study and oversaw all aspects of study implementation. AG and MB-S collected the data and DP managed the database. AG and DP performed the statistical analyses and edited the final draft. MB-S wrote the first draft of the manuscript. All authors contributed to the article and approved the submitted version.

## Conflict of interest

The authors declare that the research was conducted in the absence of any commercial or financial relationships that could be construed as a potential conflict of interest.

## Publisher’s note

All claims expressed in this article are solely those of the authors and do not necessarily represent those of their affiliated organizations, or those of the publisher, the editors and the reviewers. Any product that may be evaluated in this article, or claim that may be made by its manufacturer, is not guaranteed or endorsed by the publisher.

## References

[ref1] Alexander K. L. (2001). Prosocial Behaviors of Adolescents in Work and Family Life: Empathy and Conflict Resolution Strategies With Parents and Peers. Dissertation Abstracts International Section A: Humanities and Social Sciences, 61, 3367.

[ref2] Alonso-Ferres M. Valor-Segura I. Expósito F. (2019). Couple conflict-facing responses from a gender perspective: emotional intelligence as a differential pattern. Psychosoc. Interv. 28, 147–156. doi: 10.5093/pi2019a9

[ref3] Ambler K. Petrides K. V. Vernon P. A. (2023). Relations between a self-defeating interpersonal style and trait emotional intelligence. Personal. Individ. Differ. 203:112026. doi: 10.1016/j.paid.2022.112026

[ref4] Arenas A. V. (2014). El papel de la relación de Pareja en los contextos familiares de riesgo psicosocial [the role of the couple's relationship in family contexts of psychosocial risk]. Doctoral thesis. Universidad de Sevilla]. idUS—Depósito de investigación Universidad de Sevilla.

[ref5] Arenas A. V. Hidalgo M. V. (2016). Calidad de la relación de Pareja en familias en situación de riesgo psicosocial: un estudio descriptivo [quality of the couple relationship in families at psychosocial risk: a descriptive study]. Apunt. Psicol. 34, 231–239. doi: 10.55414/ap.v34i2-3.614

[ref6] Augusto J. M. Aguilar-Luzón M. D. C. Salguero de Ugarte M. F. (2008). El papel de la IEP y del Optimismo/pesimismo disposicional en la resolución de problemas sociales: un estudio con alumnos de trabajo social [the role of perceived emotional intelligence and dispositional optimism/pessimism in social problem solving: a study of social work students]. Electron. J. Res. Educ. Psychol. 6, 363–382. doi: 10.25115/ejrep.v6i15.1282

[ref7] Bailen N. H. Green L. M. Thompson R. J. (2019). Understanding emotion in adolescents: a review of emotional frequency, intensity, instability, and clarity. Emot. Rev. 11, 63–73. doi: 10.1177/1754073918768878

[ref8] Batool S. S. Khalid R. (2012). Emotional intelligence: a predictor of marital quality in Pakistani couples. Pak. J. Psychol. Res. 27, 65–88. Available at: https://www.researchgate.net/publication/277220120_Emotional_Intelligence_A_Predictor_of_Marital_Quality_in_Pakistani_Couples

[ref9] Bentler P. M. (2005). EQS 6.1: Structural Equations Program Manual Multivariate Software, Inc.

[ref10] Bentler P. M. Bonett D. G. (1980). Significance tests and goodness of fit in the analysis of covariance structures. Psychol. Bull. 88, 588–606. doi: 10.1037/0033-2909.88.3.588, PMID: 30383824

[ref11] Bentler P. M. Chou C. P. (1987). Practical issues in structural modeling. Sociol. Methods Res. 16, 78–117. doi: 10.1177/0049124187016001004, PMID: 37463526

[ref12] Berenguer M. (2019). Creación y validación de una escala Para la medida del desbordamiento emocional en la relación de Pareja [creation and validation of a scale for the measurement of emotional flooding in the couple relationship]. [Master's thesis, Universidad Camilo José Cela].

[ref13] Black K. A. (2000). Gender differences in adolescents’ behavior during conflict resolution tasks with best friends. Adolescence 35, 499–512. PMID: 11130594

[ref14] Blau P. M. (1964). Justice in social exchange. Sociol. Inq. 34, 193–206. doi: 10.1111/j.1475-682X.1964.tb00583.x, PMID: 37463314

[ref15] Booth A. Crouter A. C. Clements M. L. Boone-Hollday T. (Eds.) (2016). Couples in Conflict. New York, NY: Routledge.

[ref16] Braiker H. B. Kelley H. H. (1979). Conflict in the development of close relationships. In Burgess R. L. Huston T. L. (Eds.), Social exchange in developing relationships. (New York, NY: Academic Press, Inc). 135–168.

[ref17] Buedo-Guirado C. Dumitrache C. G. Rubio L. (2021). 507—stressful past events and emotional intelligence as predictors of successful aging. Int. Psychogeriatr. 33, 59–60. doi: 10.1017/S1041610221002027

[ref18] Burns T. (1973). A structural theory of social exchange. Acta Sociol. 16, 188–208. doi: 10.1177/000169937301600303, PMID: 37465489

[ref19] Byrne B. M. (1994). Structural Equation Modeling With EQS and EQS/Windows: Basic Concepts, Applications, and Programming. Thousand Oaks, CA: SAGE Publications.

[ref20] Byrne B. M. (2010). Structural Equation Modeling With AMOS. Basic Concepts, Applications, and Programming. 2nd *Edn*. New York, NY: Routledge, Taylor & Francis Group.

[ref21] Carstensen L. L. Fung H. H. Charles S. T. (2003). Socioemotional selectivity theory and the regulation of emotion in the second half of life. Motiv. Emot. 27, 103–123. doi: 10.1023/A:1024569803230

[ref22] Cascón P. (2006). Educar en y Para el conflicto [educating in and for conflict]. 6, 12–21.

[ref23] Chen Y. Peng Y. Fang P. (2016). Emotional intelligence mediates the relationship between age and subjective well-being. Int. J. Aging Hum. Dev. 83, 91–107. doi: 10.1177/0091415016648705, PMID: 27199490 PMC5442987

[ref24] Chentsova-Dutton Y. E. Tsai J. L. (2007). Gender differences in emotional response among European Americans and Hmong Americans. Cognit. Emot. 21, 162–181. doi: 10.1080/02699930600911333, PMID: 26342220

[ref25] Chester C. E. Blandon A. Y. (2016). Dual trajectories of maternal parenting stress and marital intimacy during toddlerhood: parenting stress and intimacy. Pers. Relat. 23, 265–279. doi: 10.1111/pere.12122

[ref26] Christensen A. Walczynski P. T. (1997). Conflict and satisfaction in couples. In Sternberg R. J. Hojjat M. (Eds.), Satisfaction in close relationships. (New York, NY: The Guilford Press). 249–274.

[ref27] Coccaro E. F. Zagaja C. Chen P. Jacobson K. (2016). Relationships between perceived emotional intelligence, aggression, and impulsivity in a population-based adult sample. Psychiatry Res. 246, 255–260. doi: 10.1016/j.psychres.2016.09.004, PMID: 27728868

[ref28] Cócola F. (2022). Apego, regulación emocional y funcionamiento familiar en adultos con trastornos por consumo de cocaína [attachment, emotional regulation, and family functioning in adults with cocaine use disorders]. Rev. Psicopatol. Psicol. Clín. 27, 59–72. doi: 10.5944/rppc.30820

[ref29] de Wied M. Branje S. J. T. Meeus W. H. J. (2007). Empathy and conflict resolution in friendship relations among adolescents. Aggress. Behav. 33, 48–55. doi: 10.1002/ab.20166, PMID: 17441005

[ref30] Delhom I. Gutierrez M. Lucas-Molina B. Meléndez J. C. (2017). Emotional intelligence in older adults: psychometric properties of the TMMS-24 and relationship with psychological well-being and life satisfaction. Int. Psychogeriatr. 29, 1327–1334. doi: 10.1017/S1041610217000722, PMID: 28462774

[ref31] Diener E. Sandvik E. Larsen R. J. (1985). Age and sex effects for emotional intensity. Dev. Psychol. 21, 542–546. doi: 10.1037/0012-1649.21.3.542, PMID: 37258562

[ref32] Durosini I. Triberti S. Savioni L. Sebri V. Pravettoni G. (2022). The role of emotion-related abilities in the quality of life of breast Cancer survivors: a systematic review. Int. J. Environ. Res. Public Health 19:12704. doi: 10.3390/ijerph191912704, PMID: 36232004 PMC9566755

[ref33] Else-Quest N. M. Higgins A. Allison C. Morton L. C. (2012). Gender differences in self-conscious emotional experience: a meta-analysis. Psychol. Bull. 138, 947–981. doi: 10.1037/a0027930, PMID: 22468881

[ref34] Extremera N. Fernández-Berrocal P. (2005). Perceived emotional intelligence and life satisfaction: predictive and incremental validity using the trait Meta-mood scale. Personal. Individ. Differ. 39, 937–948. doi: 10.1016/j.paid.2005.03.012, PMID: 17295973

[ref35] Fairfield B. Mammarella N. Palumbo R. Di Domenico A. (2015). Emotional Meta-memories: a review. Brain Sci. 5, 509–520. doi: 10.3390/brainsci5040509, PMID: 26569320 PMC4701025

[ref37] Fernández-Aguilar L. Ricarte J. Ros L. Latorre J. M. (2018). Emotional differences in young and older adults: films as mood induction procedure. Front. Psychol. 9:1110. doi: 10.3389/fpsyg.2018.01110 30018584 PMC6037940

[ref36] Fernández-Berrocal P. Extremera N. (2006). Emotional intelligence: a theoretical and empirical review of its first 15 years of history. Psicothema 18, 7–12. Available at: https://www.psicothema.com/pdf/3270.pdf 17295952

[ref38] Fernández-Berrocal P. Extremera N. Ramos N. (2004). Validity and reliability of the spanish modified version of the trait Meta-mood scale. Psychol. Rep. 94, 751–755. doi: 10.2466/pr0.94.3.751-755, PMID: 15217021

[ref39] Ferrer V. A. Bosch E. (2005). Introduciendo la perspectiva de género en la investigación psicológica sobre violencia de género [Introducing a gender perspective in psychological research on gender violence]. Anal. Psicol. 21, 1–10. Available at: https://revistas.um.es/analesps/article/view/27061/26251

[ref40] Fischer A. (2000). Gender and Emotion: Social Psychological Perspectives. Cambridge, UK: Cambridge University Press.

[ref41] Fishbane M. D. (2011). Facilitating relational empowerment in couple therapy. Fam. Process 50, 337–352. doi: 10.1111/j.1545-5300.2011.01364.x, PMID: 21884074

[ref42] Galindo-Domínguez H. Saínz de la Maza M. Losada Iglesias D. (2022). La inteligencia emocional en el desarrollo de estilos de resolución de conflictos en futuros educadores [Emotional intelligence in the development of conflict resolution styles in future educators]. Rev. Electrón. Interuniver. Form. Profesor. 25, 141–157. doi: 10.6018/reifop.528721

[ref43] Garaigordobil M. Machimbarrena J. M. Maganto C. (2016). Adaptación española de un instrumento Para evaluar la resolución de conflictos (Conflictalk): Datos psicométricos de fiabilidad y validez [Spanish adaptation of an instrument to assess conflict resolution (Conflictalk): psychometric data of reliability and validity]. Rev. Psicol. Clín. Niños Adolesc. 3, 59–67. Available at: https://www.revistapcna.com/sites/default/files/16-23.pdf

[ref44] Garaigordobil M. Maganto C. (2011). Empatía y resolución de conflictos durante la infancia y la adolescencia. Rev. Latinoam. Psicol. 43, 255–266. Available at: http://www.scielo.org.co/pdf/rlps/v43n2/v43n2a05.pdf

[ref45] García B. de Oliveira O. (2007). “Trabajo extradoméstico y relaciones de género: Una nueva mirada [Extradomestic work and gender relations: A new look].” in Género, familias y trabajo: Rupturas y continuidades. Desafíos para la investigación política. CLACSO, Consejo Latinoamericano de Ciencias Sociales.

[ref46] García del Castillo J. A. García del Castillo-López A. Gázquez M. Marzo J. A. (2013). La Inteligencia Emocional como estrategia de prevención de las adicciones [Emotional Intelligence as an addiction prevention strategy]. Health Addict. 13, 89–97. doi: 10.21134/haaj.v13i2.204

[ref47] García M. A. Umberson D. (2019). Marital strain and psychological distress in same-sex and different-sex couples. J. Marriage Fam. 81, 1253–1268. doi: 10.1111/jomf.12582, PMID: 31496540 PMC6731029

[ref48] Goleman D. (1996). Inteligencia Emocional. Kairós.

[ref49] Goleman D. Boyatzis R. McKee A. (2002). Primal Leadership: Realizing the Power of Emotional Intelligence. Boston, MA: Harvard Business School Press, 306.

[ref50] Gómez-Leal R. Megías-Robles A. Gutiérrez-Cobo M. J. Cabello R. Fernández-Berrocal P. (2022). Personal risk and protective factors involved in aggressive behavior. J. Interpers. Violence 37, NP1489–NP1515. doi: 10.1177/0886260520926322 32529937

[ref51] Górriz A. B. Etchezahar E. Pinilla-Rodríguez D. E. Giménez-Espert M. C. Soto-Rubio A. (2021). Validation of TMMS-24 in three Spanish-speaking countries: Argentina, Ecuador, and Spain. Int. J. Environ. Res. Public Health 18:9753. doi: 10.3390/ijerph18189753 34574687 PMC8469647

[ref52] Gottman J. M. (1993a). A theory of marital dissolution and stability. J. Fam. Psychol. 7, 57–75.

[ref53] Gottman J. M. (1993b). The roles of conflict engagement, escalation, and avoidance in marital interaction: a longitudinal view of five types of couples. J. Consult. Clin. Psychol. 61, 6–15. doi: 10.1037/0022-006X.61.1.6 8450108

[ref54] Gottman J. M. (1994). What Predicts Divorce? The Relationship Between Marital Processes and Marital Outcomes. Hillsdale, NJ: Lawrence Erlbaum Associates, Inc.

[ref55] Gottman J. M. Silver N. (2012). Siete Reglas de Oro Para Vivir en Pareja: Un Estudio Exhaustivo Sobre las Relaciones y la Convivencia [Seven Golden Rules for Living as a Couple: A Comprehensive Study on Relationships and Living Together]. Barcelona: Penguin Random House Grupo Editorial España.

[ref56] Grossman M. Wood W. (1993). Sex differences in intensity of emotional experience: a social role interpretation. J. Pers. Soc. Psychol. 65, 1010–1022. doi: 10.1037/0022-3514.65.5.1010, PMID: 8246109

[ref57] Hair J. F. Black W. C. Babin B. J. Anderson R. E. (2010). Multivariate Data Analysis. 7th *Edn*. New York, NY: Pearson.

[ref58] Hansson M. Ahlborg T. (2016). Factors contributing to separation/divorce in parents of small children in Sweden. Nordic Psychol. 68, 40–57. doi: 10.1080/19012276.2015.1071201

[ref59] Homans G. C. (1974). Social Behavior: Its Elementary Forms, Revised Edition. New York, NY: Harcourt Brace Jovanovich, 386.

[ref60] Hoyle R. H. (1995). Structural Equation Modelling: Concepts, Issues, and Applications Thousand Oaks, CA: Sage.

[ref61] Isaza L. (2011). Causas y estrategias de solución de conflictos en las relaciones de Pareja formadas por estudiantes universitarios [causes and conflict resolution strategies in couple relationships formed by university students]. Psicogente 14, 336–351. Available at: https://www.redalyc.org/articulo.oa?id=497552359009

[ref62] Işık R. A. Kaya Y. (2022). The relationships among perceived stress, conflict resolution styles, spousal support and marital satisfaction during the COVID-19 quarantine. Curr. Psychol. 41, 3328–3338. doi: 10.1007/s12144-022-02737-4, PMID: 35730053 PMC9203771

[ref63] Jardine B. B. Vannier S. Voyer D. (2022). Emotional intelligence and romantic relationship satisfaction: a systematic review and meta-analysis. Personal. Individ. Differ. 196:111713. doi: 10.1016/j.paid.2022.111713

[ref64] Johnson M. D. Horne R. M. Hardy N. R. Anderson J. R. (2018). Temporality of couple conflict and relationship perceptions. J. Fam. Psychol. 32, 445–455. doi: 10.1037/fam0000398, PMID: 29723003

[ref65] Jöreskog K. G. (1993). Testing Structural Equations Models. In Bollen K. A. Long J. S. (Eds). Thousand Oaks, CA: Sage.

[ref66] Jöreskog K. G. Sörbom D. (1981). Analysis of covariance structure. Scand. J. Stat. 8, 65–92.

[ref67] Jöreskog K. G. Sörbom D. (1984). LISREL VI. Scientific Software.

[ref68] Jöreskog K. G. Sörbom D. (1986). LISREL VI: Analysis of linear structural relationships by maximum likelihood and least squares methods. Scientific Software.

[ref69] Juarros-Basterretxea J. Herrero J. Ocampo N. Y. Rodríguez-Díaz F. J. (2022). Dyadic analysis of emotional intimate partner violence: an estimation of dyadic patterns and influencing individual, family, and couple factors. Eur. J. Psychol. Appl. Legal Context 14, 105–111. doi: 10.5093/ejpalc2022a10

[ref70] Kieslich U. Steins G. (2022). Long-term couple relationships—stress, problems and coping processes in couple counseling: insights based on five case studies with five long-term couples. Front. Psychol. 13:866580. doi: 10.3389/fpsyg.2022.866580, PMID: 36337490 PMC9632744

[ref71] Kolb B. Whishaw I. Q. Teskey G. C. (2019). An introduction to Brain and Behavior. 6th Edn. New York, NY: Worth Publishers.

[ref72] Kopystynska O. Paschall K. W. Barnett M. A. Curran M. A. (2017). Patterns of interparental conflict, parenting, and children’s emotional insecurity: a person-centered approach. J. Fam. Psychol. 31, 922–932. doi: 10.1037/fam0000343, PMID: 28795829

[ref73] Kring A. M. Gordon A. H. (1998). Sex differences in emotion: expression, experience, and physiology. J. Pers. Soc. Psychol. 74, 686–703. doi: 10.1037/0022-3514.74.3.686, PMID: 9523412

[ref74] Kuncewicz D. Kuncewicz D. Mroziński B. Stawska M. (2021). A combination of insecure attachment patterns in a relationship and its quality: the role of relationship length. J. Soc. Pers. Relat. 38, 648–667. doi: 10.1177/0265407520969896

[ref75] Kunzmann U. Kappes C. Wrosch C. (2014). Emotional aging: a discrete emotions perspective. Front. Psychol. 5:380. doi: 10.3389/fpsyg.2014.00380, PMID: 24834060 PMC4018521

[ref76] Kunzmann U. Wrosch C. (2018). Comment: the emotion–health link: perspectives from a lifespan theory of discrete emotions. Emot. Rev. 10, 59–61. doi: 10.1177/1754073917719332

[ref77] Kurdek L. A. (1994). Conflict resolution styles in gay, lesbian, heterosexual nonparent, and heterosexual parent couples. J. Marriage Fam. 56, 705–722. doi: 10.2307/352880

[ref78] Laca F. A. Alzate R. Sánchez M. Verdugo J. C. Guzmán J. (2006). Communication and conflict in young Mexican students: messages and attitudes. Conflict Resolut. Q. 24, 31–54. doi: 10.1002/crq.156

[ref79] Luque-Reca O. Pulido-Martos M. Lopez-Zafra E. Augusto-Landa J. M. (2018). The importance of emotional intelligence and cognitive style in institutionalized older adults’ quality of life. J. Gen. Psychol. 145, 120–133. doi: 10.1080/00221309.2018.1437384, PMID: 29768128

[ref80] Ma H. K. (2005). The relation of gender-role classifications to the prosocial and antisocial behavior of Chinese adolescents. J. Genet. Psychol. 166, 189–202. doi: 10.3200/GNTP.166.2.189-202 15906931

[ref81] Malik J. Heyman R. E. Smith Slep A. M. (2020). Emotional flooding in response to negative affect in couple conflicts: individual differences and correlates. J. Fam. Psychol. 34, 145–154. doi: 10.1037/fam0000584, PMID: 31393141 PMC7007326

[ref82] Marsh H. W. Grayson D. (1995). Latent variable models of multitrait-multimethod data. In Hoyle R. (Ed.), Structural Equation Modeling: Concepts, Issues and Applications. (Thousand Oaks, CA: Sage), 177–198.

[ref83] Martín J. F. (2005). Los factores definitorios de los grandes grupos de la población: Tipos, subgrupos y umbrales [the defining factors of large population groups: types, subgroups and thresholds]. Geo Crítica / Scripta Nova. Revista Electrónica de Geografía y Ciencias Sociales, IX(190). Available at: https://www.ub.edu/geocrit/sn/sn-190.htm

[ref84] Mayer J. D. Caruso D. R. Salovey P. (1999). Emotional intelligence meets traditional standards for an intelligence. Intelligence 27, 267–298. doi: 10.1016/S0160-2896(99)00016-1, PMID: 34605292

[ref85] Mayer J. D. Salovey P. (1997). What is emotional intelligence? In Salovey P. Sluyter D. (Eds.), Emotional development and emotional intelligence: Educational implications. (New York, NY: Basic Books), 3–31.

[ref86] Meléndez J. C. Delhom I. Satorres E. (2019). El poder de la inteligencia emocional sobre la resiliencia en adultos mayores [the power of emotional intelligence on resilience in older adults]. Ansied. Estrés 25, 14–19. doi: 10.1016/j.anyes.2019.01.001

[ref87] Milani A. S. Hosseini M. Matbouei M. Nasiri M. (2020). Effectiveness of emotional intelligence training program on marital satisfaction, sexual quality of life, and psychological well-being of women. J. Educ. Health Promot. 9, 1–8. doi: 10.4103/jehp.jehp_124_20.2019.01.001 32766334 PMC7377144

[ref88] Monk J. K. Ogolsky B. G. Oswald R. F. (2018). Coming out and getting Back in: relationship cycling and distress in same- and different-sex relationships. Fam. Relat. 67, 523–538. doi: 10.1111/fare.12336

[ref89] Montes-Berges B. (2009). Patrones de comunicación, diferenciación y satisfacción en la relación de Pareja: Validación y análisis de estas escalas en muestras españolas [patterns of communication, differentiation and satisfaction in the couple relationship: validation and analysis of these scales in Spanish simples]. Anal. Psicol. 25, 288–298. Available at: https://revistas.um.es/analesps/article/view/87661

[ref90] Oussi A. Hamid K. Bouvet C. (2023). Managing emotions in panic disorder: a systematic review of studies related to emotional intelligence, alexithymia, emotion regulation, and coping. J. Behav. Ther. Exp. Psychiatry 79:101835. doi: 10.1016/j.jbtep.2023.101835, PMID: 36680910

[ref91] Penberthy J. K. Chhabra D. Ducar D. M. Avitabile N. Lynch M. Khanna S.. (2018). Impact of coping and communication skills program on physician burnout, quality of life, and emotional flooding. Saf. Health Work 9, 381–387. doi: 10.1016/j.shaw.2018.02.005, PMID: 30559985 PMC6284159

[ref92] Perles F. San Martín J. Canto J. Moreno P. (2011). Inteligencia emocional, celos, tendencia al abuso y estrategias de resolución de conflicto en la pareja [Emotional intelligence, jealousy, tendency to abuse and conflict resolution strategies in couples]. Escrit. Psicol. 4, 34–43. doi: 10.5231/psy.writ.2011.0605

[ref93] Pineda D. Rico-Bordera P. Galán M. Piqueras J. A. González-Álvarez J. L. (2023). Women victims of intimate partner violence and intimate partner homicide: a typology based on victimization variables. Psychosoc. Interv. 32, 43–53. doi: 10.5093/pi2023a3, PMID: 37361632 PMC10268547

[ref94] Pineda D. Valiente R. M. Chorot P. Antonio Piqueras J. Sandín B. (2018). Invarianza factorial y temporal del Cuestionario de Regulación Emocional (ERQ) [factorial and temporal invariance of a Spanish version of the emotional regulation questionnaire (ERQ)]. Rev. Psicopatol. Psicol. Clín. 23, 109–120. doi: 10.5944/rppc.vol.23.num.2.2018.21823

[ref95] Quesada S. (2020). Empatía y Estilos de Comunicación en Pareja: Consecuencias sobre la Satisfacción en la Pareja [Empathy and Communication Styles in Couples: Implications for Couple Satisfaction] Universitat de les Illes Balears. Available at: http://dspace.uib.es/xmlui/handle/11201/152720

[ref96] Rachman S. (1966). Studies in desensitization—II: flooding. Behav. Res. Ther. 4, 1–6. doi: 10.1016/0005-7967(66)90037-4, PMID: 5929526

[ref97] Rivers S. E. Brackett M. A. Katulak N. A. Salovey P. (2007). Regulating anger and sadness: an exploration of discrete emotions in emotion regulation. J. Happiness Stud. 8, 393–427. doi: 10.1007/s10902-006-9017-2, PMID: 37315591

[ref98] Rodríguez M. Díaz D. (2013). Noviazgo: Evolucion del significado psicologico durante la adolescencia [Datin: Evolution of psychological meaning during adolescence]. Rev. Psicol. 10, 20–32.

[ref99] Rojas-Solís J. L. Romero-Méndez C. A. (2022). Violencia en el noviazgo: análisis sobre su direccionalidad, percepción, aceptación, consideración de gravedad y búsqueda de apoyo [Dating violence: analysis on its directionality, perception, acceptance, consideration of severity and support seeking]. Health Addict. 22, 132–151. doi: 10.21134/haaj.v22i1.638

[ref100] Rozen N. Aderka I. M. (2023). Emotions in social anxiety disorder: a review. J. Anxiety Disord. 95:102696. doi: 10.1016/j.janxdis.2023.102696, PMID: 36878132

[ref101] Ruiz-Mamani P. G. Cunza-Aranzábal D. F. White M. Quinteros-Zúñiga D. Jaimes-Soncco J. E. Morales-García W. C. (2022). Psychometric properties of the trait meta-mood scale for measuring emotional intelligence in peruvian students. Behav. Psychol. 30, 447–463. doi: 10.51668/bp.8322207n

[ref102] Sabo N. (2020). Emotional intelligence and conflict resolution styles: A quantitative study with practical implications [Undergraduate thesis project, Metropolia University of Aplied Sciences]. Theseus. Available at: https://urn.fi/URN:NBN:fi:amk-2020051511593

[ref103] Salguero J. M. Palomera R. Fernández-Berrocal P. (2012). Perceived emotional intelligence as predictor of psychological adjustment in adolescents: a 1-year prospective study. Eur. J. Psychol. Educ. 27, 21–34. doi: 10.1007/s10212-011-0063-8

[ref104] Salovey P. Mayer J. (1990). Emotional Intelligence. Imagin. Cogn. Pers. 9, 185–211. doi: 10.2190/DUGG-P24E-52WK-6CDG, PMID: 37463384

[ref105] Salovey P. Mayer J. D. Goldman S. L. Turvey C. Palfai T. P. (1995). Emotional attention, clarity and repair: exploring emotional intelligence using the trait Meta-mood scale. In Pennebaker J. W. (Ed.), Emotion, Disclosure and Health. (Washington, D.C.: American Psychological Association), 125–154.

[ref106] Salovey P. Stroud L. R. Woolery A. Epel E. S. (2002). Perceived emotional intelligence, stress reactivity, and symptom reports: further explorations using the trait Meta-mood scale. Psychol. Health 17, 611–627. doi: 10.1080/08870440290025812

[ref107] Schumm W. A. Nichols C. W. Schectman K. L. Grigsby C. C. (1983). Characteristics of responses to the Kansas marital satisfaction scale by a sample of 84 married mothers. Psychol. Rep. 53, 567–572. doi: 10.2466/pr0.1983.53.2.567

[ref108] Shrout M. R. Black A. E. Wilson S. J. Renna M. E. Madison A. D. Kiecolt-Glaser J. K.. (2023). How aging couples’ emotional and physiological associations change across positive, supportive, and conflictual discussions: roles of capitalization and responsive behaviors. Biol. Psychol. 177:108500. doi: 10.1016/j.biopsycho.2023.108500, PMID: 36646301 PMC10023389

[ref109] Skordoulis M. Koukounaras Liagkis M. Sidiropoulos G. Drosos D. (2020). Emotional intelligence and workplace conflict resolution: the case of secondary education teachers in Greece. Int. J. Res. Educ. Sci. 6:521. doi: 10.46328/ijres.v6i4.1224

[ref110] Smith T. W. Berg C. A. Florsheim P. Uchino B. N. Pearce G. Hawkins M.. (2009). Conflict and collaboration in middle-aged and older couples: I. age differences in agency and communion during marital interaction. Psychol. Aging 24, 259–273. doi: 10.1037/a0015609, PMID: 19485646 PMC4560488

[ref111] Sotskova A. Woodin E. M. Gou L. H. (2015). Hostility, flooding, and relationship satisfaction: predicting trajectories of psychological aggression across the transition to parenthood: hostility, flooding, and psychological aggression. Aggress. Behav. 41, 134–148. doi: 10.1002/ab.21570, PMID: 27539934

[ref112] Tanaka J. S. (1993). Multifaceted conceptions of fit in structural equation models. In Bollen K. A. Long J. S. (Eds.),Testing Structural Equation Models. (Newbury Park, CA: Sage), 10–40.

[ref113] Thibaut J. W. Kelley H. H. (1959). The Social Psychology of Groups. New York, NY: Routledge, 313.

[ref114] Totenhagen C. J. Butler E. A. Curran M. A. Serido J. (2016). The calm after the storm: relationship length as associated with couples’ daily variability. J. Soc. Pers. Relat. 33, 768–791. doi: 10.1177/0265407515597562

[ref115] Twenge J. M. Campbell W. K. Foster C. A. (2003). Parenthood and marital satisfaction: a Meta-analytic review. J. Marriage Fam. 65, 574–583. doi: 10.1111/j.1741-3737.2003.00574.x

[ref116] Vanderbilt R. R. Solomon D. H. (2022). The role of perceived resolvability in serial arguments across the lifespan. Pers. Relat. 29, 236–257. doi: 10.1111/pere.12415

[ref117] Vasconcelos P. A. Ramos C. Paúl C. Nobre P. J. (2021). Sexual conservatism and sexual satisfaction in older women: a cross-sectional mediation analysis. Clin. Gerontol. 44, 249–258. doi: 10.1080/07317115.2021.1872755, PMID: 33478374

[ref118] Veloso-Besio C. B. Cuadra-Peralta A. A. Antezana-Saguez I. Avendaño-Robledo R. Fuentes-Soto L. (2013). Relación entre inteligencia emocional, satisfacción vital, felicidad subjetiva y resiliencia en funcionarios de educación especial [relationship between emotional intelligence, life satisfaction, subjective happiness and resilience in special education officials]. Estud. Pedagóg. 39, 355–366. doi: 10.4067/S0718-07052013000200022

[ref119] Vidal L. F. Rivera Aragón S. Díaz-Loving R. Méndez Ramírez I. (2012). Elaboración de una escala de permanencia en la relación de pareja [Development of a scale of permanence in the couple relationship]. Rev. Iberoam. Diagnóst. Eval. Avalia. Psicol. 1, 199–225. Available at: https://www.redalyc.org/articulo.oa?id=459645437011

[ref120] Winardi M. A. Prentice C. Weaven S. (2022). Systematic literature review on emotional intelligence and conflict management. J. Glob. Scholars Market. Sci. 32, 372–397. doi: 10.1080/21639159.2020.1808847, PMID: 37452524

[ref121] Zeidner M. Kaluda I. (2008). Romantic love: What’s emotional intelligence (EI) got to do with it? Personal. Individ. Differ. 44, 1684–1695. doi: 10.1016/j.paid.2008.01.018

